# Antiandrogens Act as Selective Androgen Receptor Modulators at the Proteome Level in Prostate Cancer Cells* [Fn FN2]

**DOI:** 10.1074/mcp.M113.036764

**Published:** 2015-02-18

**Authors:** Greg N. Brooke, Simon C. Gamble, Michael A. Hough, Shajna Begum, D. Alwyn Dart, Michael Odontiadis, Sue M. Powell, Flavia M. Fioretti, Rosie A. Bryan, Jonathan Waxman, Robin Wait, Charlotte L. Bevan

**Affiliations:** From the ‡Androgen Signalling Laboratory, Imperial College London, London W12 0NN, UK;; §Molecular Oncology, School of Biological Sciences, University of Essex, Colchester CO4 3SQ, UK;; ¶Kennedy Institute of Rheumatology, Imperial College London, London W6 8LH, UK;; ‖Cardiff University Peking University Cancer Institute, Cardiff University School of Medicine, Cardiff CF14 4XN, UK

## Abstract

Current therapies for prostate cancer include antiandrogens, inhibitory ligands of the androgen receptor, which repress androgen-stimulated growth. These include the selective androgen receptor modulators cyproterone acetate and hydroxyflutamide and the complete antagonist bicalutamide. Their activity is partly dictated by the presence of androgen receptor mutations, which are commonly detected in patients who relapse while receiving antiandrogens, *i.e.* in castrate-resistant prostate cancer. To characterize the early proteomic response to these antiandrogens we used the LNCaP prostate cancer cell line, which harbors the androgen receptor mutation most commonly detected in castrate-resistant tumors (T877A), analyzing alterations in the proteome, and comparing these to the effect of these therapeutics upon androgen receptor activity and cell proliferation. The majority are regulated post-transcriptionally, possibly via nongenomic androgen receptor signaling. Differences detected between the exposure groups demonstrate subtle changes in the biological response to each specific ligand, suggesting a spectrum of agonistic and antagonistic effects dependent on the ligand used. Analysis of the crystal structures of the AR in the presence of cyproterone acetate, hydroxyflutamide, and DHT identified important differences in the orientation of key residues located in the AF-2 and BF-3 protein interaction surfaces. This further implies that although there is commonality in the growth responses between androgens and those antiandrogens that stimulate growth in the presence of a mutation, there may also be influential differences in the growth pathways stimulated by the different ligands. This therefore has implications for prostate cancer treatment because tumors may respond differently dependent upon which mutation is present and which ligand is activating growth, also for the design of selective androgen receptor modulators, which aim to elicit differential proteomic responses dependent upon cellular context.

Prostate tumors are dependent upon the androgen receptor (AR)^1^ for growth. The AR is a ligand-activated transcription factor that promotes prostate cancer growth through genomic and nongenomic actions. In the canonical genomic pathway, the AR regulates transcription following interaction with specific DNA sequences, termed androgen response elements, in the regulatory regions of target genes (1). More recently, it has been demonstrated that cytoplasmic AR, within minutes of activation, also stimulates kinase signaling cascades (*e.g.* ERK and PI3K) and that this nongenomic signaling is also important in proliferation (2). The first line of treatment for nonlocalized, therefore, inoperable disease is androgen blockade. This involves chemical castration to reduce testicular production of androgens and administration of antiandrogens, which bind to the AR and hold it in an inactive state.

Hormone therapy is initially successful in the majority of patients (3), but invariably fails after a median period of 13 months, growth recurs and the disease proceeds to castrate resistance (CRPC). Multiple mechanisms have been proposed to explain CRPC and much evidence exists to suggest that even in the androgen-depleted environment, the AR continues to drive growth (4). For example, mutations of the AR have been detected in 2–25% of hormone sensitive tumors and 10–40% of cases of hormone refractory disease (5). These mutations appear to be the result of selective pressure induced by the treatment itself and in some cases the mutant receptors can be activated by alternative ligands, including antiandrogens used in therapy (6). The majority of mutations identified to date cluster in the ligand binding domain (LBD) of the receptor (4) and of those that have been studied at the functional level, several appear to offer a growth advantage because of reduced ligand specificity, enhanced androgen sensitivity, or constitutive activity (6–10). Other studies have determined the responses of prostate cancer cells to various ligands of the AR and it has been demonstrated that ligand-specific gene regulation by the AR can occur (11–13).

The most frequently reported mutation, associated with prostate cancer, is a substitution of threonine to alanine at amino acid 877 (T877A). The T877A mutation appears to be more prevalent in patients who relapse following treatment with the antiandrogen hydroxyflutamide (6) and when compared with the wild-type receptor, this mutant has increased transcriptional activity in the presence of other steroid hormones, such as estradiol and progesterone and also the antiandrogens cyproterone acetate and hydroxyflutamide (14). This activation by antiandrogens is not universal, as the antiandrogen bicalutamide is able to block activity of this mutant (15). To determine the extent to which proteomic responses to androgens and antiandrogens overlap in the presence of this mutant receptor, we exposed the LNCaP prostate cancer cell line, which harbors the T877A AR variant, to the dihydrotestosterone (DHT) analog mibolerone, cyproterone acetate, hydroxyflutamide, and bicalutamide. 2-Dimensional polyacrylamide gel electrophoresis (2-DE) was used to determine protein regulation in whole cell lysates and sets of regulated proteins were compared. Characterization of the proteomic response to antiandrogen exposure will provide further insight into the phenomenon of receptor promiscuity in CRPC and also highlight future targets for therapy once antiandrogen resistance has occurred.

## EXPERIMENTAL PROCEDURES

### 

#### 

##### Cell Culture and Treatments

HeLa cells and the LNCaP prostate cancer cell line were obtained from ATCC and cultured at 37 °C, 5% CO_2_ in Dulbecco's modified Eagle's medium (DMEM) and Roswell Park Memorial Institute (RPMI) medium 1640 (Invitrogen, Strathclyde, UK) respectively, both supplemented with 2 mm l-Glutamine, 100units/ml penicillin, 100 mg/ml streptomycin (Sigma Aldrich, St Louis, MO) and 10% fetal bovine serum. The LNCaP-PHB cell line has been previously described (16) and was grown in media supplemented as above with the exception that 10% tetracycline-free fetal bovine serum was used and the cells additionally supplemented with 12 μg/ml blasticidin (Invitrogen, Carlsbad, CA), 500 μg/ml G418 (Sigma-Aldrich), and 0.3 mg/ml zeocin (Invitrogen).

Mibolerone (Perkin-Elmer, Beaconsfield, UK), cyproterone acetate (Sigma-Aldrich, Dorsett, UK), bicalutamide (Astra-Zeneca, Cheshire, UK), and hydroxyflutamide (Schering-Plough, Hertfordshire, UK) were resuspended in ethanol and stored at −20 °C until use, final concentrations were 10 nm for mibolerone and 1 μm for antiandrogens.

##### Reporter Assays

HeLa cells were grown to 60% confluence in phenol red free media containing 5% double charcoal stripped serum, in 24-well plates. After 24 h, cells were transfected using FuGENE 6 (following the manufacturers instructions) with 100 ng wild type or mutant pSV-AR, 100 ng PDM-LAC-Z-β-GAL, and 1 μg of luciferase reporter (TAT-GRE-E1B-LUC) per well. Eighteen hours post-transfection cells were treated with ligand for 24 h. Luciferase and β-galactosidase expression was quantified as previously described (14).

##### Cell Proliferation Assay

LNCaP cells were plated at a density of 10^4^ cells per well in 96-well plates in RPMI. After 16 h incubation, the wells were washed twice in phosphate buffered saline (PBS) before incubation for 48 h in phenol-red free RPMI supplemented with 5% charcoal stripped FBS, 2 mm l-Glutamine, 100units/ml penicillin, and 100 mg/ml streptomycin. Hormone was subsequently directly to the media cells incubated for a further 72 h. To measure cellular proliferation, mitochondrial dehydrogenase activity was assayed using the WST-1 reagent (Roche Applied Science Ltd, Hertfordshire, UK) as per manufacturer's instructions. Eight wells were assayed per condition in each of three independent experiments.

##### 2-Dimensional Gel Electrophoresis (2-DE)

Five samples were prepared per experimental condition. 25 cm^2^ flasks of LNCaP cells were transferred to phenol red-free RPMI supplemented with 2 mm l-Glutamine, 100units/ml penicillin, 100 mg/ml streptomycin, and 10% charcoal stripped fetal bovine serum for 48 h before exposure to hormone or ethanol. Hormone was added to the media and mixed thoroughly. Cells were incubated as above for 16 h, then placed on ice and washed twice in PBS before lysis in 200 μl isoelectric focusing buffer. Isoelectric focusing (IEF) was performed using immobilized pH gradient (IPG) strips (GE Healthcare, Amersham Biosciences, UK), of pH range 3–10 (linear). The solubilized protein sample was applied to the strips during gel rehydration, according to the manufacturer's instructions. The samples were diluted with rehydration solution containing 8 m urea, 0.5% CHAPS, 0.2% DTT, and 0.2% Pharmalyte (pH 3–10) prior to loading, total protein loaded was 250 μg in 450 μl. The strips were focused at 0.05 mA/IPG strip for 60 kVh at 20 °C. After IEF, the strips were equilibrated in 1.5 m Tris, pH 8.8, buffer containing 6 m urea, 30% glycerol, 2% SDS, and 0.01% bromphenol blue, with the addition of 1% DTT for 15 min, followed by the same buffer with the addition of 4.8% iodoacetamide for 15 min. SDS-PAGE was performed using 12% T, 2.6% C separating polyacrylamide gels without a stacking gel, using the Iso-Dalt system (GE Healthcare). The second-dimension separation was carried out overnight at 20 mA/gel, 15 °C and was stopped when the bromphenol blue dye-front was ∼1 cm from the bottom of the gels.

##### Protein Spot Imaging and Gel Image Analysis

The dye front on each gel was removed using a scalpel blade and the gels fixed in 40% methanol 10% acetic acid for 1 h. Gels were then incubated in Sypro-ruby protein stain (GE Healthcare) overnight then washed in distilled water for 30 min. Stained gels were scanned using a Typhoon phosphorimager on fluorescent mode (GE Healthcare) and analytical images were analyzed using PDQuest version 8 (Bio-Rad, Hemel Hempstead, UK). After detection of spots, the gels were aligned, landmarked and matched. Gels were then placed into the appropriate experimental class and differential analysis performed. The student *t* test was used to detect all spots that differed significantly between the control and exposed groups (*p* < 0.05), all significantly different spots were then checked manually to eliminate any artifactual differences because of gel pattern distortions and inappropriately matched or badly detected spots.

##### Mass Spectrometry

Proteins spots of interest were excised manually from gels and subjected to in gel digestion with trypsin as described previously (17). Tandem mass spectra were recorded using a Q-Tof spectrometer (Waters, Manchester, UK) interfaced to a Waters CapLC capillary chromatograph. Samples were dissolved in 0.1% formic acid and injected onto a 300 μm × 5 mm Pepmap C18 column (LC Packings, Amsterdam, NL) and eluted with an acetonitrile/0.1% formic acid gradient. The capillary voltage was set to 3500 V. A survey scan over the m/z range 400–1300 was used to identify protonated peptides with charge states of 2, 3, or 4, which were automatically selected for data-dependent MS/MS analysis, and fragmented by collision with argon. The resulting product ion spectra were transformed onto a singly charged m/*z* axis using a maximum entropy method (MaxEnt3, Waters) and proteins were identified by correlation of uninterpreted spectra to entries in SwissProt/TrEMBL, using ProteinLynx Global Server (Version 2.2, Waters). The database was created by merging the FASTA format files of SwissProt (2012_09 release), TrEMBL, and their associated splice variants (1,768,175 entries at the time of searching). No taxonomic or protein mass and pI constraints were applied. One missed cleavage per peptide was allowed, and the initial mass tolerance window was set to 100 ppm. For further confirmation of the identifications, the spectra were also searched against the NCBI nr database (4,496,228 sequences as of January 2007) using Mascot v.2.2 (www.matrixscience.com) (18). For an identification to be considered valid we required two or more peptides were identified, that the peptide score was significant (typically greater than 55 (*p* < 0.05)), and that manual interpretation confirmed agreement between spectrum and peptide sequence. In addition, Mascot searches of all spectra were performed against a randomized version of the NCBI database using the same parameters as in the main search. In no case did this search retrieve more than a single peptide, and in all instances, the peptide score was below the 0.05 significance level.

##### Immunoblotting

Cells were treated and incubated as indicated, washed in PBS and harvested by cell scraping. Cells were pelleted (1200 rpm, 4 min), lysed in 9 m Urea and protein concentrations determined using a modified Bradford Assay (19) (Bio-Rad, Hemel Hempstead, UK). SDS-PAGE and immunoblotting was performed as described previously (20) using the following antibodies: Anti-AR (N-20, Santa Cruz Biotechnology, Dallas, TX); Anti-Flag (M-2, Sigma Aldrich) and β-actin (AC-15, Abcam, Cambridge, UK).

##### Real-Time Quantitative PCR

Cells were grown in hormone-depleted media for 72 h and treated with ligand for 16 h. RNA was extracted using TRI-Sure reagent (Bioline, Taunton, MA) and a DNase step included (Thermo Scientific, Leicestershire, UK). Reverse transcription was performed using the nanoScript RT Kit (Primer Design, Southampton, UK). Alterations in gene expression were quantitifed using a qPCR (Roche LightCycler 96, Roche, IN).

##### Comparison of AR Crystal Structures

Crystal structures retrieved from the Protein Data Bank were superposed using a “secondary structure matching” algorithm implemented in the program Superpose (21) within the CCP4 suite (22). Figures were prepared using PyMol (23).

## RESULTS

### 

#### 

##### Confirmation of Growth and Transcriptional Responses to Androgen and Antiandrogens in the LNCaP Cell Line

It is well documented that AR containing the T877A substitution has reduced ligand specificity, allowing it to be activated by a range of compounds that repress or do not fully activate the wild-type receptor (4). In order to determine the extent of this activation for the ligands under investigation, we carried out transcriptional activation assays comparing wild-type AR and AR_T877A_ in AR negative HeLa cells, and cell growth assays in LNCaP cells, in the presence of the synthetic androgen mibolerone (a nonmetabolisable analog of the natural ligand DHT, with the same relative binding affinity (24, 25)), the partial agonists cyproterone acetate and hydroxyflutamide, and the pure antagonist, bicalutamide. The relative binding affinities of the latter three ligands for wild-type AR are between 1 and 6% as compared with DHT (24, 26) (supplemental Fig. S1).

**Fig. 1.**
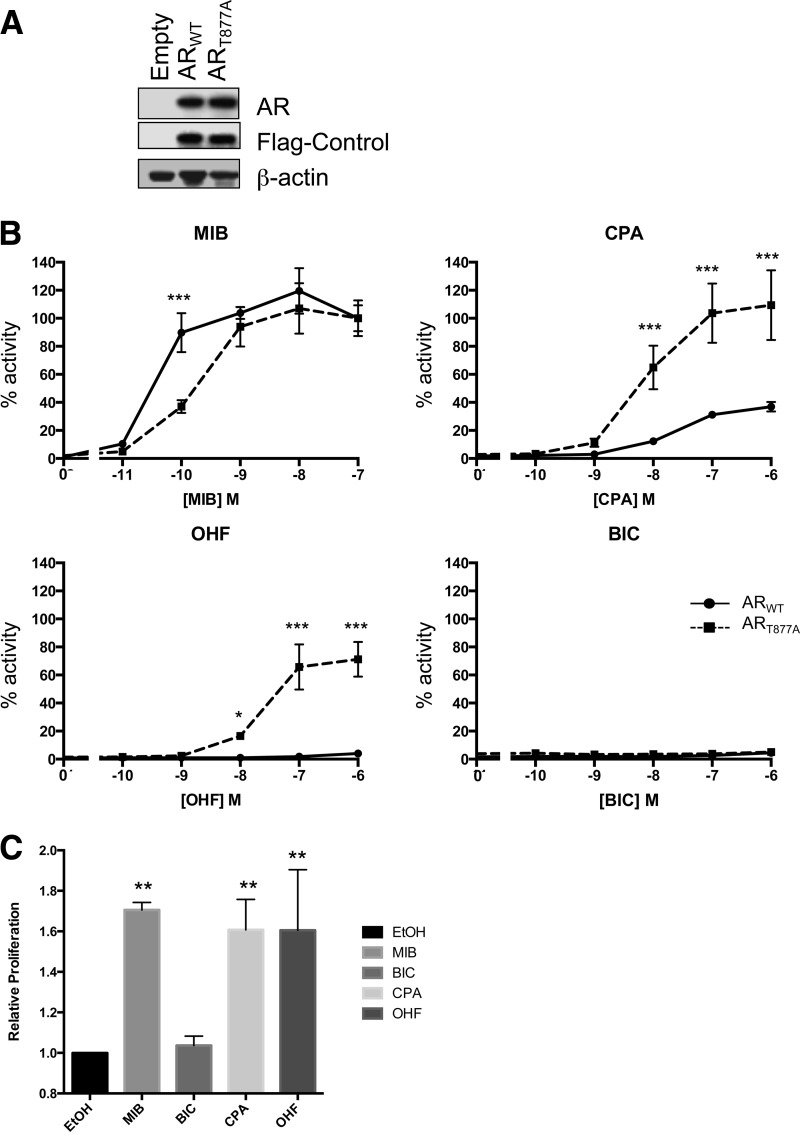
**Effect of T877A substitution on AR activity and cell growth in the presence of androgens and antiandrogens.** *A*, Expression levels of the transfected wild-type and T877A mutant androgen receptors. Cells were transfected with expression vector for either the wild-type AR or AR_T877A_ and a flag-tagged control plasmid (to control for transfection efficiency). The empty lane refers to cells transfected with empty plasmid. Cells were lysed and proteins visualized by Western blotting. Blots were probed with anti-AR and re-probed with antiflag and β-actin antibodies. *B*, HeLa cells were transiently transfected with expression vector for either the wild-type AR or AR_T877A_ mutant, TAT-GRE-E1B-LUC luciferase reporter and a β-galactosidase expression vector. Cells were exposed to ligand for 16 h. Luciferase activities were normalized for β-galactosidase activity and expressed as a percentage of the wild-type receptor activity in the presence of 10^−7^ m MIB. Data presented are the mean of three independent experiments performed in duplicate ± S.D. *C*, LNCaP cells were exposed to 10 nm mibolerone, or 1 μm antiandrogen, for 72 h and proliferation measured using WST-1 assays. Representative data of three individual experiments is presented, bars are means + S.D. of eight replicates. ANOVA + Tukey: * *p* < 0.05, ** *p* < 0.005, *** *p* < 0.001. Significant differences are comparison between AR wild-type and AR T877A in *B*, and between EtOH and ligands in *C*,. ETOH, ethanol; MIB, mibolerone; CPA, cyproterone acetate; OHF, hydroxyflutamide; BIC, bicalutamide.

Immunoblotting confirmed equal expression of the wild-type and mutant AR in transfected cells (Fig. 1*A*). Wild-type AR and AR_T877A_ were activated to a similar extent in the presence of the higher concentrations of mibolerone (Fig. 1*B*) and in keeping with previous studies, AR_T877A_ was found to have higher levels of activity when compared with wild-type AR in the presence of cyproterone acetate and hydroxyflutamide, whereas bicalutamide did not activate either of the receptors. To investigate whether this altered transcriptional profile correlated with cell growth driven by AR_T877A_, proliferation assays were performed. The LNCaP cell line was cultured in the presence of 10 nm Mibolerone or 1 μm antiandrogen for 72 h. As expected, the growth observed in response to the different ligands correlated with transcriptional response (Fig. 1*C*); mibolerone, cyproterone acetate, and hydroxyflutamide all induced similar increases in growth that were significantly greater than that observed in untreated control cells, whereas no change in growth was evident following treatment with bicalutamide.

##### Regulation of Protein Features Following Exposure to Androgens and Antiandrogens in LNCaP Cells

**Fig. 2.**
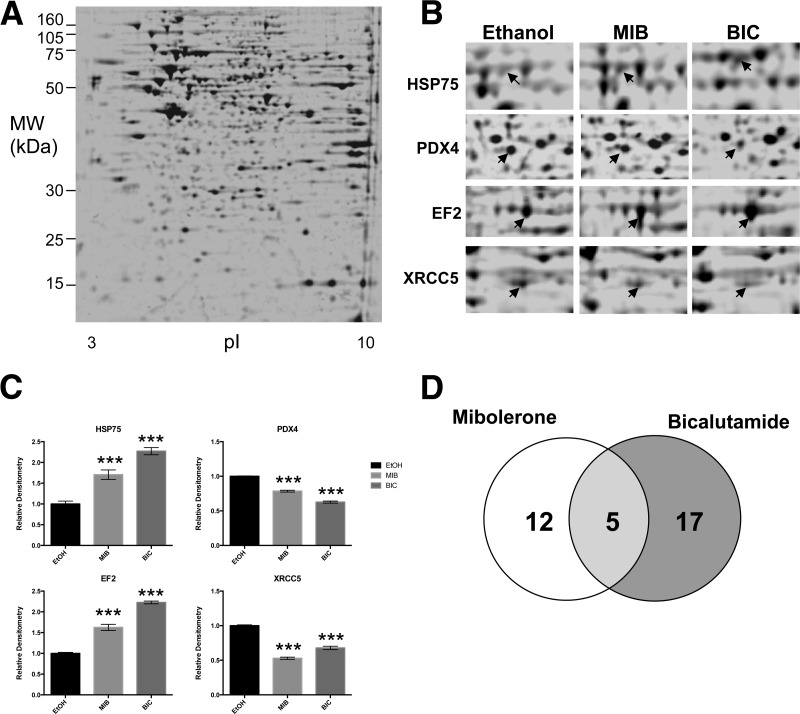
**2-DE profile of cell extract from LNCaP exposed to mibolerone and bicalutamide.** LNCaP cells in culture were exposed to 10 nm mibolerone, or 1 μm antiandrogen, for 16 h. *A*, Filtered, inverted, fluorescent gel image of gel used as master gel. Cell lysates were subjected to IEF over a linear range of pI 3–10 and 12% PAGE on 24 cm gels. Differential regulation of protein features was detected using PDQuest software using student *t* test (*p* < 0.05). *B*, regulation of protein features by exposure to androgen and antiandrogen. Images are filtered inverted fluorescent gel images of individual gels; regulated protein features are highlighted with arrows. *C*, graphical representation of regulated protein features. Data shown represents mean ± S.D. of fluorescence intensity recorded for each gel, *n* = 5 (samples from independent studies). *D*, Venn diagram of numbers of regulated protein features. ETOH, ethanol; MIB, mibolerone; BIC, bicalutamide. T-Test: *** *p* < 0.001. Significant differences are comparison between EtOH and other ligands.

The first set of 2-DE experiments was intended to characterize the “pure” androgen- and antiandrogen-related responses at the proteome level. Cells were treated with vehicle (Ethanol, EtOH), mibolerone or bicalutmide for 16 h. Five individual protein samples were subjected to 2-DE per treatment and between 419 and 997 protein features were detected across the gels. Of these features, between 320 and 698 were matched across the data set and 286 were matched to all 15 gels (Fig. 2*A*). PDQuest analysis detected 34 protein features regulated in comparison to ethanol controls, using student *t* test with 95% confidence limits, with examples of proteins being both up and down regulated and some with multiple isoforms being regulated (Table I and examples in Fig. 2*B* and 2*C*). Of the regulated proteins, 12 were regulated only by mibolerone, 17 by bicalutamide, and five were regulated by both (Fig. 2*D*).

**Table I:** Proteins regulated by androgen and antiandrogens in the LNCaP prostate cancer cell line LNCaP cells in culture were exposed to 10 nm mibolerone, 1 μm antiandrogen, or equivalent volume of ethanol for 16 hours. Protein lysates were subjected to 2-DE as described under “Experimental Procedures” and expression profiles compared to the ethanol control gels. The identities of individual protein spots were identified by mass spectrometry, excluding identities where fewer than two peptides were matched. MIB, mibolerone; CPA, cyproterone acetate; OHF, hydroxyflutamide; BIC, bicalutamide. *, This isoform of protein DJ1 was included due to an acceptably high MASCOT score on the single peptide identified and the conclusive identification of the adjacent isoform. The spectrum for this peptide is given in Supplemental Figure 2.

Protein	UniProtKB/Swiss-Prot	Unique Peptides identified	Mass (Da)	pI	% coverage	Regulation	Peptide Sequences	Actual mass	Parent charge	Delta AMU	Mascot Identity Score	Previous proteomic studies
**Regulation by one ligand**												
**Regulation by mibolerone**												
Fructose-bisphosphate aldolase A, ALDOA	P04075	2	39421	8.5	7	↑ MIB	(K)ADDGRPFPQVIK(S)	1,341.73	3	0.0289	43.7038	
							(K)GILAADESTGSIAK(R)	1,331.71	2	0.0176	43.9277	
Glyceraldehyde-3-phosphate dehydrogenase, GAPDH	P04406	4	36053	8.7	19	↑ MIB	(R)GALQNIIPASTGAAK(A)	1410.79	2	0.0042	43.3937	Up with androgen (27)
							(K)LVINGNPITIFQER(D)	1612.89	2	−0.0030	43.4891	
							(K)VIHDNFGIVEGLMTTVHAITATQK(T)	2610.37	4	0.0214	42.0445	
							(R)VVDLMAHMASKE(-)	1361.64	2	0.0097	43.6173	
Protein disulphide isomerase, P4HB	P07237	4	57118	5.0	17	↑ MIB	(K)MDSTANEVEAVK(V)	1,308.60	2	0.0089	43.2936	Up with androgen (12)
							(R)NNFEGEVTKENLLDFIK(H)	1,009.02	3	0.0104	42.8724	
							(R)TGPAATTLPDGAAAESLVESSEVAVIGFFK(D)	1,934.46	3	−0.0220	41.5042	
							(K)VDATEESDLAQQYGVR(G)	1,779.83	2	0.0014	43.2064	
ATP synthase alpha chain, ATP5A1	P25705	8	59752	9.4	21	↓ MIB	(R)EVAAFAQFGSDLDAATQQLLSR(G)	1,337.15	3	−0.0075	42.6079	
							(R)ILGADTSVDLEETGR(V)	1,574.79	2	0.0102	43.6403	
							(K)LKEIVTNFLAGFEA(-)	1,550.83	2	−0.0007	43.3614	
							(K)QGQYSPmAIEEQVAVIYAGVR(G)	1,324.14	2	−0.0077	42.5195	
							(R)TGAIVDVPVGEELLGR(V)	1,623.88	2	0.0006	43.2758	
							(R)TGAIVDVPVGEELLGRVVDALGNAIDGKGPIGSK(A)	1,315.82	4	0.0108	40.8962	
							(K)TSIAIDTIINQK(R)	1,315.74	2	0.0075	44.0358	
							(R)VVDALGNAIDGKGPIGSK(A)	1,709.46	3	−0.4724	46.1755	
Prohibitin, PHB	P35232	6	29805	5.7	36	↓ MIB	(K)AAELIANSLATAGDGLIELR(K)	1,996.68	2	−0.40030	45.7758	Down with androgen (12)
							(K)AAIISAEGDSK(A)	1,060.56	2	0.01648	44.3077	
							(K)FGLALAVAGGVVNSALYNVDAGHR(A)	1,370.25	3	0.00730	42.4301	
							(R)FVVEKAEQQKK(A)	1,332.76	2	0.01518	43.7535	
							(K)KAAIISAEGDSK(A)	1,188.66	2	0.02428	44.0912	
							(R)VLPSITTEILK(S)	1,212.47	2	−0.26790	45.2466	
Transketolase, TKT	P29401	6	67879	7.6	15	↓ MIB	(K)ILATPPQEDAPSVDIANIR(M)	1,018.85	2	−0.2093	44.2177	
							(R)MPSLPSYK(V)	937.384	2	−0.07394	42.8301	
							(K)NMAEQIIQEIYSQIQSK(K)	1,037.82	3	−0.1855	44.7493	
							(K)NSTFSEIFKK(E)	1,199.51	2	−0.1114	44.5603	
							(R)SVPTSTVFYPSDGVATEK(A)	1,883.74	2	−0.1749	45.1061	
							(R)VLDPFTIKPLDR(K)	1,412.70	2	−0.1078	43.9023	
Protein DJ-1, PARK7	Q99497	5	19891	6.5	40	↓ MIB	(K)DGLILTSR(G)	873.33	2	−0.1643	45.5546	Up with androgen (12)
							(K)EGPYDVVVLPGGNLGAQNLSESAAVK(E)	2583.32	3	−0.0026	42.1269	
							(K)GAEEmETVIPVDVmRR(A)	1862.43	3	−0.4600	46.1251	
							(R)GPGTSFEFALAIVEALNGK(E)	1920.01	2	0.0130	42.9763	
**Regulation by partially activating anti-androgens**												
Electron transfer flavoprotein subunit alpha, ETFA	P13804	10	35080	8.8	45	↑ CPA	(K)DPEAPIFQVADYGIVADLFK(V)	1,207.12	2	0.0039	42.6515	Up with androgen (27)
							(K)GLLPEELTPLILATQK(Q)	1,734.98	2	−0.0303	43.2673	
							(R)GTSFDAAATSGGSASSEK(A)	1,629.71	2	0.0030	43.3319	
							(K)IVAPELYIAVGISGAIQHLAGMK(D)	1,366.30	3	−0.0069	42.5188	
							(K)LLYDLADQLHAAVGASR(A)	1,811.97	3	0.0157	43.1861	
							(K)SDRPELTGAK(V)	1,072.57	2	0.0156	44.5317	
							(K)SGENFKLLYDLADQLHAAVGASR(A)	1,474.27	4	0.0121	42.2274	
							(K)SPDTFVR(T)	820.433	2	0.0251	44.0125	
							(K)TIVAINKDPEAPIFQVADYGIVADLFK(V)	1,946.57	3	−0.0004	41.5116	
							(R)VAAKLEVAPISDIIAIK(S)	1,750.08	3	0.0170	42.6259	
Heterogeneous nuclear ribonucleoprotein A2, HNRNPA2	P22626	2	37430	9.1	9	↑ CPA	(R)GFGFVTFDDHDPVDKIVLQK(Y)	1,276.15	3	−0.0019	42.4561	
							(K)YHTINGHNAEVR(K)	1,409.70	3	0.0234	43.4488	
Heterogeneous nuclear ribonucleoprotein L, hnRNP L	P14866	4	64132	7.1	7	↑ CPA	(K)ISRPGDSDDSR(S)	1,203.56	2	0.0131	43.9155	Down with androgen (40)
							(K)SKPGAAMVEMADGYAVDR(A)	1,898.87	3	0.0201	43.0884	
							(K)SKPGAAMVEMADGYAVDR(A)	1,898.86	3	0.0084	43.0820	
							(R)VFNVFCLYGNVEK(V)	1,587.79	2	0.0099	43.5862	
Succinyl-COa:3-ketoacid-coenzyme A transferase 1, OXCT1	P55809	5	56159	7.6	14	↓ CPA	(K)AVFDVDKKK(G)	1,048.61	2	0.0178	43.8419	
							(K)DGSVAIASKPR(E)	1,099.61	2	0.0066	44.1472	
							(K)GLTAVSNNAGVDNFGLGLLLR(S)	1,100.10	2	−0.0323	42.7747	
							(K)GMGGAMDLVSSAK(T)	1,254.57	2	0.0103	43.3927	
							(R)QYLSGELEVELTPQGTLAER(I)	1,232.12	2	−0.0080	42.7107	
**Regulation by bicalutamide**												
RuvB-like 2, RUVBL2	Q9Y230	3	51158	5.6	10	↑ BIC	(R)AVLIAGQPGTGK(T)	1,110.65	2	0.0135	42.8538	
							(K)EYQDAFLFNELK(G)	1,515.75	2	0.0216	43.6769	
							(R)TQGFLALFSGDTGEIKSEVR(E)	1,154.11	3	0.0118	42.7224	
Heat Shock Protein 75kDa, HSP75	Q12931	4	80113	8.0	11	↑ BIC	(R)GVVDSEDIPLNLSR(E)	1,512.66	2	−0.1235	44.4245	
							(R)SIFYVPDMKPSMFDVSR(E)	1,049.75	3	−0.1992	45.4958	
							(R)YESSALPSGQLTSLSEYASR(M)	1,144.85	2	−0.1695	45.0706	
							(K)YSNFVSFPLYLNGR(R)	1,675.68	2	−0.1535	44.9867	
Glutathione S-transferase Mu 3, GSTM3	P21266	2	26561	5.4	15	↓ BIC	(K)FKLDLDFPNLPYLLDGK(N)	1,007.09	3	0.0170	42.9383	
							(K)LTFVDFLTYDILDQNR(I)	1,972.02	2	0.0216	42.8312	
Lactoglutathione lyase, GLO1	Q04760	4	20779	5.3	27	↓ BIC	(K)DFLLQQTMLR(V)	1,279.54	2	−0.1168	43.8582	
							(R)FEELGVK(F)	820.37	2	−0.0599	44.0207	
							(K)FSLYFLAYEDKNDIPK(E)	1,961.85	2	−0.1308	44.0524	
							(R)VLGMTLIQK(C)	1,017.49	2	−0.0992	44.4769	
Methylmalonate-semialdehyde dehydrogenase, ALDH6A1	Q02252	2	57840	8.5	5	↓ BIC	(K)AISFVGSNK(A)	921.41	2	−0.0779	43.3670	
							(R)VNAGDQPGADLGPLITPQAK(E)	1960.88	2	−0.1390	43.3905	
ATP synthase subunit beta, ATP5B	P06576	3	56561	5.3	12	↓ BIC	(R)AIAELGIYPAVDPLDSTSR(I)	1,987.07	2	0.0412	42.9732	
							(K)SLQDIIAILGmDELSEEDKLTVSR(A)	1,690.39	3	0.0168	41.9413	
							(K)VLDSGAPIKIPVGPETLGR(I)	1,918.10	3	0.0095	43.5095	
							(K)VLDSGAPIKIPVGPETLGR(I)	1,918.13	3	0.0452	43.9718	
Peroxiredoxin 4, PDX4	Q13162	2	30541	6.2	8	↓ BIC	(R)IPLLSDLTHQISK(D)	1,463.89	3	0.0504	43.1435	
							(R)LVQAFQYTDK(H)	1,211.64	2	0.0169	43.4573	
Ran-specific GTPase-activating protein, RANBP1	P43487	2	23311	5.3	10	↓ BIC	(R)FLNAENAQK(F)	1,033.44	2	−0.0796	44.4553	
							(K)TLEEDEEELFK(M)	1,380.53	2	−0.1010	44.8707	
***Regulation by multiple ligands***												
**Regulation by antiandrogens only**												
ATP-dependent RNA helicase, DDX3X	O00571	7	73246	7.2	15	↑ CPA>↑OHF	(K)DLLDLLVEAK(Q)	1,127.65	2	0.0058	43.9529	
							(R)LEQELFSGGNTGINFEK(Y)	1,881.91	2	0.0016	42.9234	
							(R)QSSGASSSSFSSSR(A)	1,360.59	2	0.0044	43.6023	
							(K)QYPISLVLAPTR(E)	1,356.78	2	0.0068	43.6832	
							(R)SFLLDLLNATGKDSLTLVFVETK(K)	1,523.38	3	−0.0065	42.1569	
							(K)SPILVATAVAAR(G)	1,167.71	2	0.0157	42.6834	
							(R)VGSTSENITQK(V)	1,162.59	2	0.0084	43.8810	
Far upstream element-binding protein 2, FBP-2	Q92945	5	73116	8.3	11	↑ BIC, CPA, OHF	(K)AINQQTGAFVEISR(Q)	1,532.80	2	0.0053	43.5492	
							(K)IGGDAATTVNNSTPDFGFGGQKR(Q)	1,309.10	3	−0.0075	42.4608	
							(R)IINDLLQSLR(S)	1,183.72	2	0.0258	43.3738	
							(R)QLEDGDQPESK(K)	1,244.58	2	0.0233	43.9370	
							(R)TSMTEEYRVPDGMVGLIIGR(G)	1,255.11	3	0.0123	42.5643	
Fumarate hydratase, FH	P07954	6	54638	9.1	23	↑ CPA, OHF	(R)AIEMLGGELGSK(I)	1,219.62	2	0.0098	43.7994	
							(R)IEYDTFGELKVPNDKYYGAQTVR(S)	1,705.34	4	0.0077	41.9537	
							(K)IPVHPNDHVNK(S)	1,268.69	3	0.0314	43.5480	
							(R)IYELAAGGTAVGTGLNTR(I)	1,762.91	2	−0.0131	43.3331	
							(K)SQSSNDTFPTAMHIAAAIEVHEVLLPGLQK(L)	1,219.60	4	−0.0252	41.0147	
							(R)THTQDAVPLTLGQEFSGYVQQVK(Y)	1,545.26	3	−0.0185	42.2817	
Bifunctional purine biosynthesis protein, PURH	P31939	4	64617	6.6	10	↓ CPA, OHF	(M)APGQLALFSVSDK(T)	1,331.72	2	0.0072	43.9252	
							(M)APGQLALFSVSDKTGLVEFAR(N)	1,205.19	3	0.0140	42.7224	
							(K)NGQVIGIGAGQQSR(I)	1,383.72	2	0.0008	43.7900	
							(K)TVASPGVTVEEAVEQIDIGGVTLLR(A)	1,552.37	3	−0.0045	42.0801	
RNA binding protein (isoform 2), DJ-1	Q99497	1*	19891	6.7	14	↓ OHF,CPA	(R)GPGTSFEFALAIVEALNGKEVAAQVK(A)	2645.40	3	−0.0081	41.8983	
Glyoxalase domain-containing protein 4, GLOD4	Q9HC38	5	34794	5.4	26	↓ OHF >↓CPA	(K)GGVDHAAAFGR(I)	1,056.43	2	−0.0767	44.9969	
							(K)ILTPLVSLDTPGK(A)	1,352.80	2	0.0094	35.0961	
							(K)LGNDFMGITLASSQAVSNAR(K)	1,066.97	2	−0.0345	42.8228	
							(K)TMVGFGPEDDHFVAELTYNYGVGDYK(L)	1,939.32	3	0.0201	33.1869	
							(K)VTLAVSDLQK(S)	1,072.62	2	0.0087	36.4748	
**Regulation in common between mibolerone and antiandrogen**												
Glutamate dehydrogenase 1, GLUD1	P00367	4	61379	8.0	15	↑ MIB, CPA, OHF	(R)DSNYHLLMSVQESLER(K)	1,935.93	3	0.0295	42.8738	Up with androgen (27)
							(K)ELEDFKLQHGSILGFPK(A)	1,957.05	3	0.0203	42.9447	
							(K)GFIGPGIDVPAPDMSTGER(E)	1,930.90	2	−0.0122	43.1061	
							(R)YSTDVSVDEVK(A)	1,240.60	2	0.0183	43.6324	
Catalase, CAT	P04040	2	59757	7.4	8	↑ MIB, CPA	(K)ADVLTTGAGNPVGDKLNVITVGPR(G)	1,363.27	3	−0.0087	42.3940	
							(R)FSTVAGESGSADTVRDPR(G)	1,850.89	3	0.0152	43.0490	
Elongation factor 2, EEF2	P13639	9	95340	6.9	20	↑ BIC >↑MIB, CPA, OHF	(R)ALLELQLEPEELYQTFQR(I)	1,218.96	3	−0.1895	43.8637	Up with androgen (12, 27, 40)
							(K)ARPFPDGLAEDIDKGEVSAR(Q)	1,142.07	3	0.0033	42.6392	
							(K)AYLPVNESFGFTADLR(S)	1,798.91	2	0.0242	35.0065	
							(K)DGAGFLINLIDSPGHVDFSSEVTAALR(V)	1,800.22	3	−0.1872	43.6065	
							(K)EGIPALDNFLDKL(-)	1,443.77	2	0.0104	43.6075	
							(R)NMSVIAHVDHGK(S)	1,322.69	3	0.0479	35.3428	
							(K)STAISLFYELSENDLNFIK(Q)	1,203.10	2	−0.0079	34.4948	
							(R)VFSGLVSTGLK(V)	1,106.66	2	0.0214	43.7057	
							(R)WLPAGDALLQMITIHLPSPVTAQK(Y)	1,615.42	3	0.0091	33.9005	
Multifunctional protein ADE2, PAICS	P22234	2	47080	7.4	11	↑ CPA, MIB	(R)IKAEYEGDGIPTVFVAVAGR(S)	1,046.56	2	0.0040	42.8197	
							(K)TKEVYELLDSPGK(V)	739.89	2	0.0056	43.4142	
Triosephosphate isomerise, TPIS	P60174	5	30791	6.9	27	↑ CPA, OHF > ↑MIB	(R)HVFGESDELIGQK(V)	1,457.75	3	0.0319	43.7497	Up with androgen (27)
							(K)QSLGELIGTLNAAK(V)	1,413.78	2	−0.0051	43.6821	
							(K)SNVSDAVAQSTR(I)	1,233.61	2	0.0182	43.6687	
							(K)TATPQQAQEVHEK(L)	1,465.72	2	0.0004	43.2650	
							(K)VTNGAFTGEISPGMIK(D)	1,636.79	2	−0.0206	43.2389	
X-ray repair cross-complementing protein 5, XRCC5	P13010	2	82707	5.6	5	↓ BIC>↓MIB	(R)DDEAAAVALSSLIHALDDLDMVAIVR(Y)	1,738.37	3	−0.0102	41.8361	Down with androgen (12, 40)
							(K)EEASGSSVTAEEAK(K)	1,393.64	2	0.0240	43.3201	
Peroxidoredoxin 2, PRDX2	P32119	6	21892	5.9	35	↓ BIC, MIB, OHF	(K)EGGLGPLNIPLLADVTR(R)	1,733.93	2	−0.0349	43.2539	
							(R)KEGGLGPLNIPLLADVTR(R)	1,862.05	2	−0.0169	43.0449	
							(K)LGCEVLGVSVDSQFTHLAWINTPR(K)	1,698.29	3	−0.0627	42.3009	
							(R)LSEDYGVLKTDEGIAYR(G)	1,927.97	3	0.0127	43.0081	
							(R)QITVNDLPVGR(S)	1,210.66	2	−0.0055	43.3476	
							(K)TDEGIAYR(G)	923.44	2	0.0083	42.4983	
Serine hydroxymethyltransferase,	P34897	4	55995	8.8	11	↓ MIB, BIC	(R)AMADALLER(G)	1,004.53	2	0.0345	44.3892	
SHMT2							(R)ISATSIFFESMPYK(L)	1,635.78	2	−0.0060	43.1923	
							(K)TGLIDYNQLALTAR(L)	1,547.83	2	−0.0045	43.5249	
							(R)VVDFIDEGVNIGLEVK(S)	1,744.92	2	−0.0001	43.4153	
**Regulation in opposition**												
Splicing factor 35kDA subunit, U2AF1	Q01081	2	27872	9.1	11	↑CPA, ↓ OHF	(M)AEYLASIFGTEKDK(V)	1,612.79	2	−0.0128		
							(R)NPQNSSQSADGLR(C)	1,372.65	2	0.0187	43.9884	
Creatine kinase B-type, CKB	P12277	7	42645	5.5	23	↑MIB, ↓BIC	(K)GGNMKEVFTR(F)	1,153.58	2	0.0286	43.3005	Up with androgen (12)
							(R)GTGGVDTAAVGGVFDVSNADR(L)	1,963.93	2	0.0065	42.7353	
							(K)LAVEALSSLDGDLAGR(Y)	1,585.86	2	0.0277	43.6743	
							(R)LEQGQAIDDLMPAQK(-)	1,671.78	2	−0.0353	43.3532	
							(R)LGFSEVELVQMVVDGVK(L)	1,863.99	2	0.0247	43.0735	
							(R)LGFSEVELVQMVVDGVKLLIEMEQR(L)	1,892.49	3	−0.0050	41.6557	
							(K)LLIEMEQR(L)	1,046.54	2	−0.0055	44.2711	
Pyruvate kinase, isozymes M1/M2, PKM	P14618	6	57938	8.2	16	↑MIB, ↓OHF	(K)FGVEQDVDMVFASFIR(K)	1,874.89	2	0.0043	43.1840	
							(K)GSGTAEVELKK(G)	1,117.61	2	0.0106	44.1767	
							(K)GVNLPGAAVDLPAVSEK(D)	1,635.89	2	0.0041	43.2004	
							(K)IYVDDGLISLQVK(Q)	1,461.82	2	0.0123	43.5367	
							(R)RFDEILEASDGIMVAR(G)	1,836.92	3	0.0178	43.0317	
							(R)SVETLKEMIK(S)	1,192.65	2	0.0108	43.4793	

**Fig. 3.**
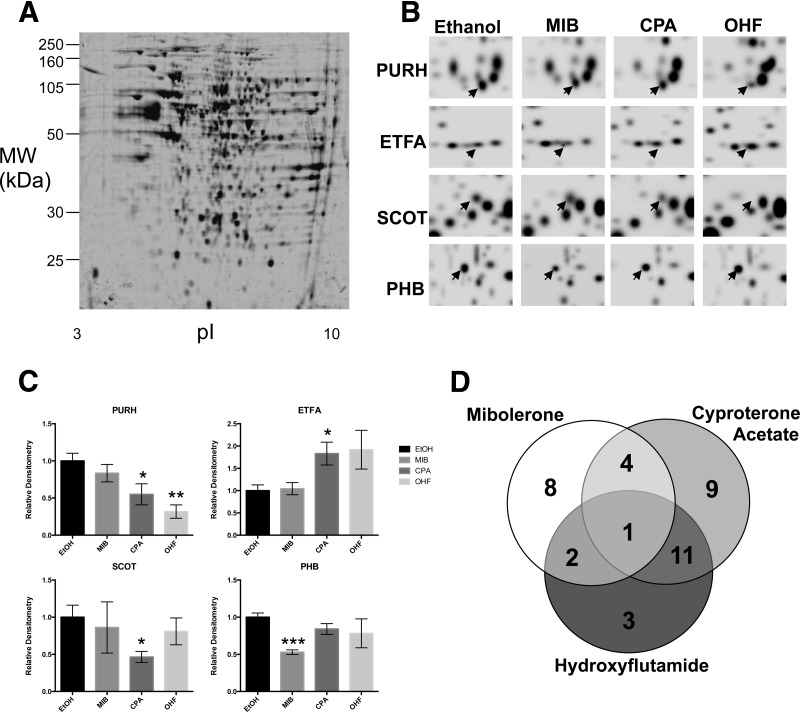
**2-DE profile of cell extract from LNCaP exposed to mibolerone and antiandrogens.** LNCaP cells in culture were exposed to 10 nm mibolerone, or 1 μm antiandrogen, for 16 h. *A*, Filtered, inverted, fluorescent gel image of gel used as master gel. Cell lysates were subjected to IEF over a linear range of pI 3–10 and 12% PAGE on 24 cm gels. Differential regulation of protein features was detected with PDQuest software using student *t* test (*p* < 0.05). *B*, regulation of protein features by exposure to androgen and antiandrogen. Images are Gaussian, inverted, fluorescent, gel images of individual gels; regulated protein features are highlighted with arrows. *C*, graphical representation of regulated protein features. Data shown represents mean ± S.D. of fluorescence intensity recorded for each gel, *n* = 5 (samples from independent studies). *D*, Venn diagram of numbers of regulated protein features. ETOH, ethanol; MIB, mibolerone; CPA, cyproterone acetate; OHF, hydroxyflutamide. T-Test: * *p* < 0.05, **p < 0.005, *** *p* < 0.001. Significant differences are comparison of EtOH and other ligands.

We next determined the extent to which exposure of the LNCaP cells to the partial agonists cyproterone acetate and hydroxyflutamide correlated with the responses to pure androgen or antiandrogen observed in the first experiment. Again, five protein samples were processed per condition (vehicle, mibolerone, cyproterone acetate, and hydroxyflutamide). The 2D gels contained between 360 and 589 protein features, and in total 241 features were matched across all gels (Fig. 3*A*). PDQuest analysis detected 38 protein features regulated in comparison to ethanol controls, using student *t* test with 95% confidence limits (Fig. 3*B* and 3*C*). Of these, 15 were regulated by mibolerone, 25 by cyproterone acetate, and 17 by hydroxyflutamide (Fig. 3*D* and Table I). Overlap between groups was greatest for features regulated by both hydroxyflutamide and cyproterone acetate, with 12 proteins being commonly regulated. Five proteins were commonly regulated between cyproterone acetate and mibolerone treated cells, whereas three were regulated in common between hydroxyflutamide and mibolerone. Only one protein feature was shown to be commonly regulated between all three exposure groups.

##### Protein Identities of Regulated Features

**Fig. 4.**
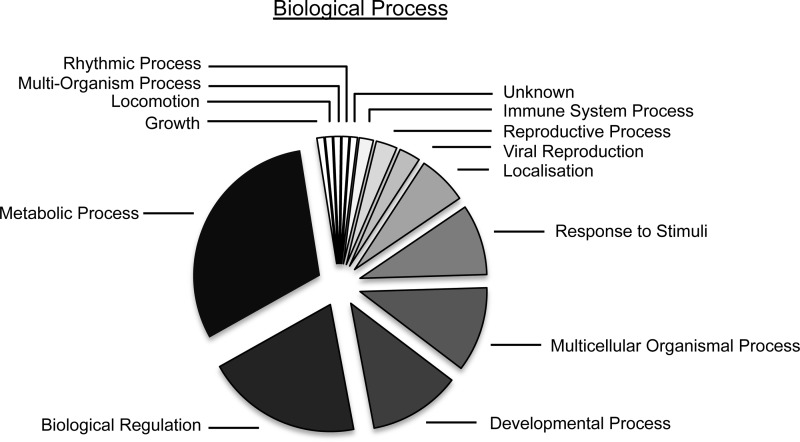
**Gene Ontology analysis of proteins regulated by the androgen receptor.** Biological processes were assigned using Scaffold Viewer (Proteome Software Portland, OR).

Mass spectrometry of regulated protein features resulted in identification of a total of 36 proteins, excluding hits where fewer than two peptides were positively identified (Table I). Proteins were submitted to Gene Ontology analysis (Scaffold) to identify key processes/functions regulated by the AR pathway. Similar to previous studies (*e.g.* (27)), the largest ontology grouping found to be regulated by the AR was Metabolic Processing (Fig. 4).

Proteins regulated by mibolerone were associated with a wide spectrum of functions and pathways, including cell cycle regulation (*e.g.* prohibitin), protein folding (*e.g.* protein disulfide isomerise 6), ATP synthesis (*e.g.* ATP-synthase alpha chain), and gene transcription (*e.g.* Protein DJ-1). Proteins regulated in common between mibolerone and the antiandrogens were included in many of these same pathways and included the antioxidant defense enzymes catalase and peroxidoredoxin 2 (Table I). Generally, proteins down-regulated by bicalutamide tended to be involved in metabolic or protein synthesis pathways (*e.g.* lactoglutathione lyase and Methylmalonate-semialdehyde dehydrogenase) or antioxidant defense (*e.g.* peroxidoredoxin 4).

##### The AR Targets Are Predominantly Regulated at the Post-transcriptional Level

**Fig. 5.**
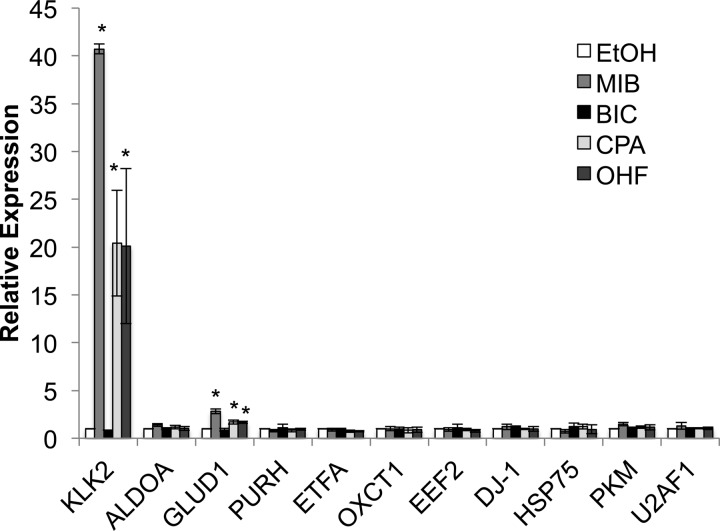
**The AR target proteins are predominantly regulated at the post-transcriptional level.** LNCaP cells were grown in steroid-depleted media for 72 h and treated with ligand for 16 h. RNA was harvested, reverse transcriped, and qPCR performed to investigate alterations in relative gene expression. Gene expression was normalized to *L19* expression. Mean of four repeats ± 1sd. T-Test * *p* < 0.05.

To investigate whether the target proteins are regulated at the transcriptional level, LNCaP cells were treated with ligand for 16 h, RNA harvested, and qPCR performed for a selection of the protein-encoding genes identified (Fig. 5). As a positive control, we confirmed mibolerone, cyproterone acetate, and hydroxyflutamide induction of the known AR target *kallikrein 2* (*KLK2*). No significant change in expression was evident for the targets, with the exception of *glutamate dehydrogenase* (*GLUD1*), which was found to be up-regulated threefold in response to mibolerone and weakly upregulated in response to cyproterone acetate snd hydroxyflutamide. It therefore appears that the majority of alterations in the proteome in response to androgen receptor activation/inhibition, at this early time point, are at the post-transcriptional level.

##### The Androgen Down-regulated Target Prohibitin: A Suppressor of Cell Growth

**Fig. 6.**
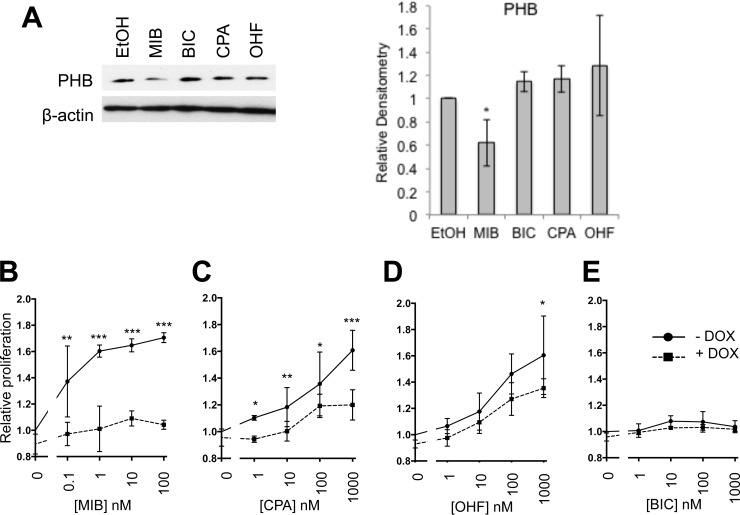
**Prohibitin selectively blocks androgen induced prostate cancer growth.** *A*, LNCaP cells were grown in hormone-depleted medium for 48 h and treated with 10 nm mibolerone or 1 μm antiandrogen, or equivalent volume of ethanol for 16 h. Protein lysates were separated by Western blotting and visualized using immunodetection and densitometry performed (mean of three independent repeats ± 1 S.D.). T-Test * *p* < 0.05. (*B–D*) the LNCaP-PHB cell line was grown in hormone-depleted medium for 72 h, treated with ligand for 96 h and proliferation assessed using WST1 assays. Mean of three independent replicates ± 1 S.D. ANOVA * *p* < 0.05, ** *p* < 0.005, *** *p* < 0.001. Significant differences are comparison of EtOH and other ligands in a, and comparison of − and + DOX in b-e.

Androgen signaling is a key driver of prostate cancer growth and as such, factors regulated by the AR are likely to be important in proliferation. An example of this is Prohibitin (PHB), which we have demonstrated to be androgen-regulated and to regulate LNCaP proliferation (data herein and (28)). PHB was found to be down-regulated in response to androgen, with little change evident in response to cyproterone acetate, hydroxyflutamide, or bicalutamide (Fig. 6*A*). We were interested to see if PHB could block LNCaP growth activated not only by androgen but also by cyproterone acetate and hydroxyflutamide. To perform this experiment, we utilized the LNCaP cell line stably transfected with the *PHB* gene under the control of the doxycycline promoter (29). Cells were plated in hormone-depleted media and treated with concentration ranges of mibolerone, bicalutamide, hydroxyflutamide, or cyproterone acetate ± doxycycline and proliferation assessed after 4 days. As expected, increasing concentrations of mibolerone, cyproterone acetate, and hydroxyflutamide promoted cell growth (Fig. 6*B*–6*D*), whereas bicalutamide had no effect on proliferation (Fig. 6*E*). Over-expression of PHB was found to significantly block the growth promoting effects of mibolerone with no significant increase in growth evident at any concentration of mibolerone (ANOVA, *p* > 0.05). In contrast, PHB was less efficient at inhibiting the growth promoting effects of hydroxyflutamide and cyproterone acetate (41 and 67% inhibition of AR activity, respectively, compared with 94% in presence of MIB) suggesting that the inhibitory effects of PHB are dependent upon the ligand driving growth (Fig. 6*C*–6*D*).

##### The Different Ligands Are Associated with Different Conformations in the AR AF-2 and BF-3 Interaction Domains

**Fig. 7.**
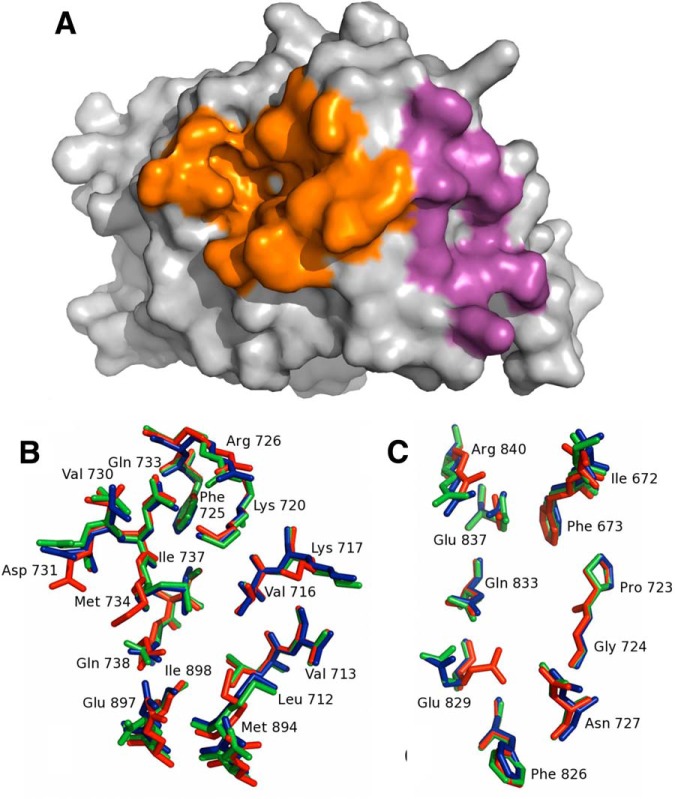
**The ligands promote different conformations in the AF-2 and BF-3 surfaces of the AR ligand binding domain.** *A*, Surface representation of AR T877A (PDB accession number 1I38) with the AF-2 and BF-3 regions colored orange and purple, respectively; *B*, the AF-2 region in the superposed structures of the androgen receptor T877A variant in complex with DHT (PDB 1I38, red) (61), cyproterone acetate (PDB 2oz7, blue) (62) and hydroxyflutamide (PDB 2ax6, green) (63). *C*, the same superposition showing residues in the BF-3 region (61–63).

To investigate potential mechanisms by which AR_T877A_ promotes ligand-dependent alterations in the proteome of LNCaP cells, we undertook detailed analysis of the available crystal structures of the AR_T877A_ ligand-binding domain in the presence of DHT (PDB accession number 1i38), cyproterone acetate (2oz7), or hydroxyflutamide (2ax6). The AF-2 and BF-3 regions of the AR are known interaction sites for cofactors and hence our analysis focused on these regions (Fig. 7*A*) (30–32).

The AF-2 surface of the AR ligand-binding domain consists of a hydrophobic cleft with charged clamp residues (Lys 720 and Glu 897) positioned at either end of the groove (14, 33). Alpha-helical motifs found in coactivators bind to this groove and form interactions with the highlighted residues (Fig. 7*B*) (30, 34). The majority of residues within the AF-2 region are similar in position in all three structures (Fig. 7*B* and 7*C*). However, the side chain positions of residues Met734, Asp731, and Met894 are similar for cyproterone acetate and hydroxyflutamide, but differ to that evident for the DHT complex. The shift in the Met894 position in the DHT complex in particular results in the side chain aligning within the groove, significantly reducing its size. The charge clamp residue Glu897 exhibits significant variability, with a different orientation in the hydroxyflutamide complex compared with that in the other two structures.

The BF3 domain of the AR is an allosteric pocket that has also been demonstrated to be important in receptor function and protein interactions (30, 32). In the case of the BF3 domain, fewer differences are apparent between the three structures although the side chain of Glu 829 occupies a different rotamer in the DHT complex compared with the other structures, with Arg 840 being differently positioned in all structures (Fig. 7*C*). In summary, the conformation of the AF-2 and BF-3 surfaces are most similar when the receptor is in complex with SARMs cyproterone acetate and hydroxyflutamide, however, differences in conformation are apparent for all ligands investigated.

## DISCUSSION

Resistance of prostate cancer to hormone therapy is a common occurrence in patients exposed to long-term treatment. Multiple mechanisms have been proposed to explain therapy relapse, including mutation of the AR resulting in constitutively or promiscuously active receptor. The reported frequency of AR mutation in recurrent prostate cancer varies greatly between studies, ranging from 10–50%, but incidence does appear to correlate with therapy resistance (4, 5, 35). One mutation detected in the AR of prostate cancer patients is a substitution of threonine to alanine at amino acid 877 (6), which is also present in the LNCaP prostate cancer cell line. In this study, we investigated the proteomic response of the LNCaP cell line in response to an androgen, the activating antiandrogens cyproterone acetate and hydroxyflutamide and bicalutamide, which remains antagonistic to LNCaP growth (36). The synthetic DHT analog mibolerone was used throughout the study because DHT is rapidly metabolized and inactivated in LNCaP cells (37); studies using androgen analogs have shown a high degree of overlap with DHT response demonstrating that the action of these synthetic ligands is comparable with the natural ligand (38).

The AR_T877A_ variant is known to be activated by several antiandrogens, including cyproterone acetate and hydroxyflutamide, and thus we have used it to determine the early proteomic responses to such ligands *versus* the response to androgen. Using WST1 proliferation assays (succinate dehydrogenase activity as a surrogate for proliferation) we confirmed that mibolerone and the antiandrogens cyproterone acetate and hydroxyflutamide promote growth of LNCaP cells. We note that our proteomic results demonstrated that the AR regulates a number of proteins involved in metabolism, however, the expression of succinate dehydrogenase was not found to be regulated in response to any of the ligands tested and our WST1 results correlate with similar proliferation studies that have utilized alternative methods to assess cell growth (*e.g.* (15, 39)).

This study has demonstrated that different ligands largely regulate different subsets of proteins, although some degree of overlap was detected between the two partial agonists and mibolerone. Interestingly, more than half of the mibolerone-regulated proteins identified here have been previously shown to be androgen regulated in proteomic studies of the LNCaP cell line (12, 27, 40, 41). Of note, our study identified fewer androgen target proteins than these previous studies, which may be explained by the shorter treatment time (16 h as opposed to 48–72 h) resulting in fewer indirect androgen protein targets being significantly regulated. In contrast, 80% of proteins regulated by cyproterone acetate and/or hydroxyflutamide, were not identified in these previous studies, supporting the supposition that these ligands promote an alternative proteomic response compared with the cognate ligand. The overlapping responses between androgen and the activating antiandrogens are of particular importance because they may therefore represent truly agonistic responses of the liganded receptor. Unsurprisingly, the largest differences were detected between cells exposed to the agonist mibolerone and the pure antiandrogen, bicalutamide. Following bicalutamide treatment, the majority of responsive proteins were down-regulated. These included proteins involved in protein synthesis, glycolysis, and cell signaling, such as lactoglutathione lyase and Ran specific GTPase activating protein. Exceptions to this observation included 75 kDa heat shock protein (HSP75), also known as TRAP1 (tumor necrosis factor receptor-associated protein-1), which was up-regulated by bicalutamide. TRAP1 has antiapoptotic functions and plays a role in multidrug resistance in colorectal carcinoma (42). The up-regulation of TRAP1 in response to bicalutamide may therefore be a cellular stress response induced by the antiandrogen.

Far upstream element-binding protein 2 (FBP2) was up-regulated in the presence of all of the antiandrogens tested, suggesting that this protein is regulated as part of an inhibitory response. The strongest response was to the pure antagonist bicalutamide, whereas an intermediate response was observed in the presence of the partial agonists hydroxyflutamide and cyproterone acetate. FBP2 is involved in AU-rich element (ARE)-mediated decay of mRNA species. This post-transcriptional regulation is important in physiological cellular proliferation and is a process that has been found to be deregulated in cancer (43). Additionally, EEF2 was regulated by all the various treatments used. Functioning as a protein elongation factor, EEF2 is regulated by a specific kinase EF2K, which is under the regulation of mTOR (44), indicating that the mTOR pathway is a point of commonality in all AR responses and therefore may have an important role in androgen regulated cell growth. The EEF2 protein is inactivated by phosphorylation, and inactivation of EF2K is increased by mTOR activity in breast cancer cells (45). Other groups have shown that up-regulation of mTOR activity occurs following androgen stimulation of LNCaP cells and in androgen independent variants of the LNCaP cell line (46–48). Our data identified regulation of two EEF2 isoforms, one of which was up-regulated in cyproterone acetate and hydroxyflutamide exposed cells, whereas the second was up-regulated in the presence of all treatments. It may therefore be the case that EEF2 levels were generally up-regulated in response to treatment, indicating a need for increased translation in all exposed cells irrespective of the nature of the treatment, whereas the modification may be a specific response to certain ligands via mTOR regulation of EEF2 kinase.

Our 2D gels showed two close lying spots, each significantly down-regulated, one by mibolerone, the other by cyproterone acetate and hydroxyflutamide, which were both identified as DJ-1. DJ-1 has been previously shown to increase AR activity by abrogating binding of the inhibitory histone deacetylase complex (49) and expression is up-regulated in several cancers, including prostate cancer (50, 51). The differential regulation of isoforms of DJ-1 by pure agonist *versus* partial agonist again suggests that there is not total overlap between the responses to the various ligands used. A previous study of DJ-1 in LNCaP cells found that protein, but not mRNA levels were increased after 48 h of treatment with mibolerone and hydroxyflutamide (52). We also found no change in the mRNA levels following treatments. This observation confirms the importance of data obtained at the proteomic level, which would not be apparent from transcript based studies and suggests that in addition to regulation of total protein levels, regulation of the isoforms of DJ-1 occurs, perhaps by post-translational modification. Of the 10 targets investigated by qPCR, only GLUD1 was regulated at the transcriptional level. This therefore suggests that the majority of early targets identified in this study are regulated by the nongenomic action of the AR. Nongenomic AR signaling is known to occur within minutes of activation and is mediated by the cytoplasmic AR, which activates kinase signaling cascades such as mTOR and PI3K (2).

The ability of steroid receptors to accept a variety of steroidal and nonsteroidal compounds as ligands is not unique to the AR. The transcriptional response of estrogen receptor α when stimulated by a range of alternative ligands has been investigated (53). Synthetic ligands for the estrogen receptor exhibit tissue specific agonist- or antagonist-like activities, and are thus termed “selective estrogen receptor modulators” (SERMs) (54, 55). Further, microarray studies of estrogen receptor α-mediated gene expression have demonstrated a spectrum of responses following exposure to estradiol, the SERMs tamoxifen and raloxifene and the pure antiestrogen ICI 182,780 (53). Our data indicates that this may also be true at the protein level for AR_T877A_. Each ligand promotes regulation of a specific set of proteins, which overlap to incorporate the proteins commonly regulated between various ligands. Frequently the magnitude of response for each protein was found to vary according to ligand and several of the proteins regulated by one ligand also showed regulation by the others, although not to a statistically significant degree.

In terms of mechanism, the difference in the level of response is likely because of the range of possible conformational changes in the receptor structure when bound to the different ligands. Analysis of available crystal structures of the AR_T877A_ ligand binding domain in complex with DHT, cyproterone acetate, or hydroxyflutamide identified important differences in the orientation of residue side chains that form the AF-2 and BF3 domains. In agreement with the finding that the antiandrogens cyproterone acetate and hydroxyflutamide had the greatest proteomic overlap, generally the conformation of the AF-2 and BF3 surfaces induced by these ligands was also similar and differed to that induced by DHT.

The BF3 domain of the AR is an allosteric pocket that has recently been demonstrated to be the site of interaction for the cochaperone Bag-1L and has received much interest as a novel site for therapeutic targeting (30, 32, 56). The AF-2 coactivator interaction groove consists of a hydrophobic cleft with charge-clamp residues (Lys720 and Glu897) situated at either end (14, 33). Coactivator interaction motifs can be broadly separated into two categories: LxxLL-type motifs and FxxLF-type motifs (where *x* = any amino acid). The AR preferentially interacts with the latter, because phenylalanine-rich motifs form electrostatic interactions with both charge-clamp residues whereas leucine-rich motifs only form hydrogen bonds with Lys720 (57). Because Glu897 is located on helix 12, which acts as a lid to the ligand-binding pocket, the positioning of this residue is greatly dependent upon the ligand bound. Indeed, the crystal structure analysis demonstrated that this residue adopts a different conformation for each of the ligands investigated. The AF-2 residues Met734, Asp731 and Met894 are also known to be important in coactivator interaction motif binding. The orientation of these was similar in the cyproterone acetate and hydroxyflutamide complexes and differed to that induced by DHT. Such conformational changes likely in turn affect protein-protein interactions with accessory proteins. We therefore hypothesize that the differences in protein expression identified here are as a result of ligand-specific receptor conformations, which promote different complex formations, subsequently affecting target regulation.

It follows that a mutant receptor activated by an antiandrogen may not elicit the same cellular responses as a wild type receptor activated by androgen. This implies that, in prostate cancer, recurrent tumors with a mutant receptor may behave differently depending on the mutation present and the ligands available to stimulate growth. Supporting evidence for this comes from our studies of the putative tumor suppressor prohibitin. In the presence of mibolerone, prohibitin is down-regulated. This suggests that loss of prohibitin is important in androgen-induced growth, in accordance with our previous data (29). Cyproterone acetate and hydroxyflutamide only weakly reduced prohibitin levels and hence we believe that loss of prohibitin is less important for growth induced by these ligands. In support of this, exogenous expression of prohibitin was significantly more potent at inhibiting androgen-induced growth compared with cyproterone acetate/hydroxyflutamide-induced growth.

Recently, second generation antiandrogens, such as ARN-509 and Enzalutamide, have entered the clinic or trials (58). This study is also likely to have relevance to these antiandrogens because AR mutations have also been associated with resistance to these therapies. For example, the AR mutation resulting in F876L has been associated with ARN-509 and Enzalutamide failure and identified in the plasma of CRPC patients (59, 60). We therefore conclude that the AR promotes differential changes in the proteome dependent upon the activating ligand and/or the mutation present and these changes appear to have a bearing on growth.

## Supplementary Material

Supplemental Data

## Footnotes

^1^ The abbreviations used are:

AR: androgen receptor
2-DE: 2-dimensional gel electrophoresis
AR_T877A_: androgen receptor variant with T877A substitution
SERM: selective estrogen receptor modulator
SARM: selective androgen receptor modulator
RPMI: Roswell Park Memorial Institute medium 1640
DMEM: Dulbecco's modified Eagle's medium
DHT: di-hydrotestosterone
CPA: cyproterone acetate
OHF: hydroxyflutamide
ETOH: ethanol
MIB: mibolerone
BIC: bicalutamide
HDAC: histone deacetylase
KHSRP: K homology splicing regulatory protein
HSP75: 75 kilodalton heat shock protein
EEF2: elongation factor 2.
