# Supplementary material for: Antiandrogens Act as Selective Androgen Receptor Modulators at the Proteome Level in Prostate Cancer Cells
Source: Mol Cell Proteomics. 2015 Feb 18;14(5):1201–16. doi: 10.1074/mcp.M113.036764 (PMC4424393; doi:10.1074/mcp.M113.036764)
Supplement: Supplemental Data [file supp_14_5_1201__index.html]

Antiandrogens Act as Selective Androgen Receptor Modulators at the Proteome Level in Prostate Cancer Cells — Proteomic Responses to SARMs — Supplemental Data 

# Antiandrogens Act as Selective Androgen Receptor Modulators at the Proteome Level in Prostate Cancer Cells

## Supplemental Data

**Files in this Data Supplement:**

- Supplemental Figure 1
- Supplemental Figure 2 - Supplemental Figure 2. Spectrum for DJ-1 peptide
