# Supplementary material for: Antiandrogens Act as Selective Androgen Receptor Modulators at the Proteome Level in Prostate Cancer Cells
Source: Mol Cell Proteomics. 2015 Feb 18;14(5):1201–16. doi: 10.1074/mcp.M113.036764 (PMC4424393; doi:10.1074/mcp.M113.036764)
Supplement: Supplemental Data [file supp_M113.036764_Brooke_Suppl_Fig_1.pdf]

Supplemental Figure 1, Brooke et al.

**Androgens**

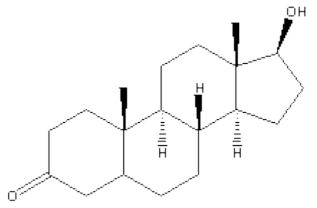

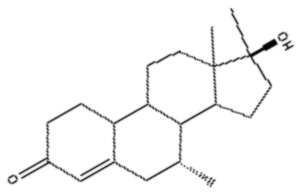

Dihydrotestosterone
(DHT)

RBA = 100 (24)

Mibolerone
(MIB)

RBA = 100 (25)

**Steroidal anti-androgen**

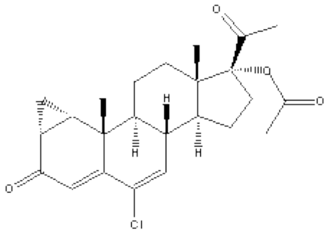

Cyproterone Acetate
(CPA)

RBA = 2.2 (26)

**Non-steroidal anti-androgen**

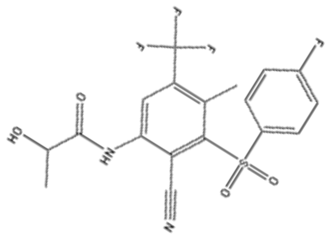

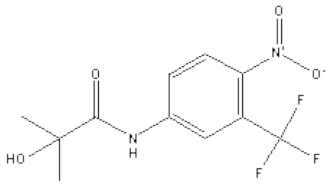

Bicalutamide
(BIC)

RBA = 6.36 (24)

Hydroxyflutamide
(OHF)

RBA = 1 (24)

**Supplemental Figure 1.** Chemical structures and relative binding affinities for ligands used. Relative binding affinities are for wild-type AR relative to R1881.
