# Supplementary material for: Antiandrogens Act as Selective Androgen Receptor Modulators at the Proteome Level in Prostate Cancer Cells
Source: Mol Cell Proteomics. 2015 Feb 18;14(5):1201–16. doi: 10.1074/mcp.M113.036764 (PMC4424393; doi:10.1074/mcp.M113.036764)
Supplement: Supplemental Data [file supp_M113.036764_mcp.M113.036764-2.pptx]

## Slide 1
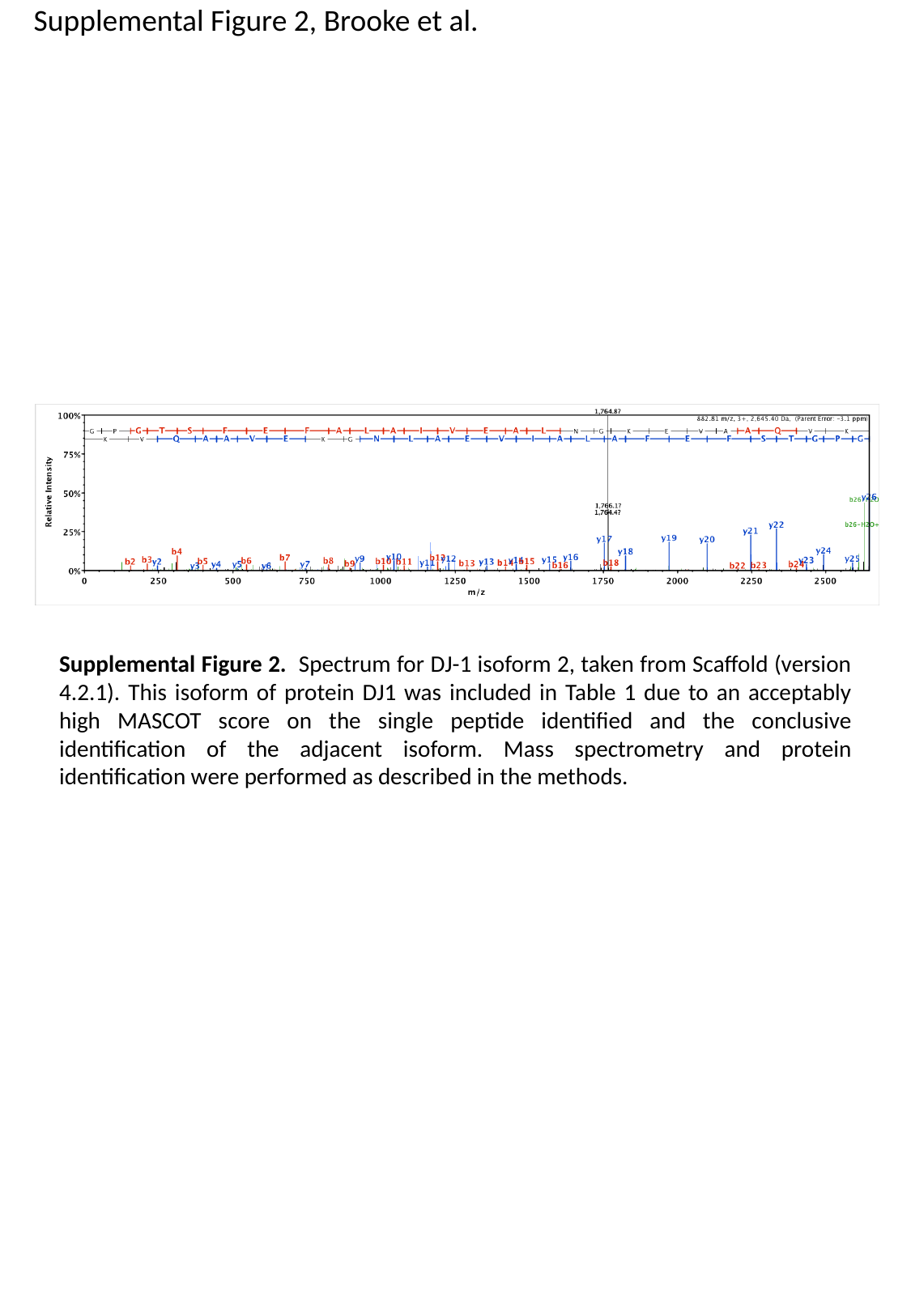

Supplemental Figure 2, Brooke et al.
Supplemental Figure 2. Spectrum for DJ-1 isoform 2, taken from Scaffold (version 4.2.1). This isoform of protein DJ1 was included in Table 1 due to an acceptably high MASCOT score on the single peptide identified and the conclusive identification of the adjacent isoform. Mass spectrometry and protein identification were performed as described in the methods.
